# Opposite effects of cefoperazone and ceftazidime on S-ribosylhomocysteine lyase/autoinducer-2 quorum sensing and biofilm formation by an *Escherichia coli* clinical isolate

**DOI:** 10.3892/mmr.2014.2540

**Published:** 2014-09-05

**Authors:** HUI-QING SHI, FENG-JUN SUN, JIAN-HONG CHEN, XIAO-LAN YONG, QIAN-YI OU, WEI FENG, PEI-YUAN XIA

**Affiliations:** 1 Department of Pharmacy, Southwest Hospital, Third Military Medical University, Chongqing 400038, P.R. China; 2 Department of Clinical Pharmacy, General Hospital of Chengdu Military Region, Chengdu, Sichuan 610083, P.R. China

**Keywords:** biofilm, cefoperazone, ceftazidime, *Escherichia coli*, S-ribosylhomocysteine lyase/autoinducer-2

## Abstract

To investigate the effects of subminimum inhibitory concentrations of cephalosporins on bacterial biofilm formation, the biofilm production of 52 *Escherichia (E.) coli* strains was examined following treatment with cephalosporin compounds at 1/4 minimum inhibitory concentrations (MICs). Ceftazidime (CAZ) inhibited biofilm formation in seven isolates, while cefoperazone (CFP) enhanced biofilm formation in 18 isolates. Biofilm formation of *E. coli* E42 was inhibited by CAZ and induced by CFP. Therefore, using reverse transcription-polymerase chain reaction, the expression of the biofilm-modulating genes of this isolate was investigated. To monitor the production of the autoinducer of quorum sensing in *E. coli*, autoinducer-2 (AI-2) production was detected by measuring the bioluminescence response of *Vibrio harveyi* BB170. Antisense oligonucleotides (AS-ODNs) targeting S-ribosylhomocysteine lyase (*luxS*) inhibited the expression of the *luxS* gene in *E. coli*. CAZ at 1/4 MIC reduced *luxS* mRNA levels and the production of AI-2, whereas CFP at 1/4 MIC had the opposite effect. AS-ODNs targeting *luxS* significantly decreased the aforementioned inhibitory effects of CAZ and the induction effects of CFP on *E. coli* biofilm formation. Therefore, biofilm formation by the *E. coli* clinical isolate E42 was evoked by CFP but attenuated by CAZ at sub-MICs, via a *luxS*/AI-2-based quorum sensing system.

## Introduction

Bacterial biofilms are a polymeric matrix produced by the bacterial cells embedded within it. The biofilm and cells together constitute a structured community that is a common cause of numerous persistent infections (1). Furthermore, such a community enables certain bacteria to control their individual gene expression via quorum sensing (QS), by detecting autoinducers secreted by themselves or their neighbors (2).

Protected within the biofilm, the bacteria are more resistant to antibiotics than their planktonic forms (3). An important example is *Escherichia (E.) coli*, a commonly encountered pathogen that is often responsible for community- and hospital-acquired infections. Isolates of *E. coli* that are harbored within the biofilm are often resistant to antibiotic treatment (4).

Emerging evidence strongly suggests that antibiotics at sub-minimum inhibitory concentrations (MICs) may nonetheless interfere with bacterial functions. These effects may have clinical relevance as bacteria are commonly exposed to sub-MICs of antibiotics at a certain period, particularly at the beginning and end of the treatment (5).

Third-generation cephalosporins, a class of β-lactam antibiotics, are widely used in the treatment of bacterial infections caused by gram-negative bacteria, such as *E. coli*. Examples of cephalosporins include ceftazidime (CAZ), cefoperazone (CFP), ceftriaxone and cefotaxime. Several studies have demonstrated that CAZ at sub-MICs inhibited QS in *Pseudomonas aeruginosa* and *Burkholderia pseudomallei*, thereby decreasing the synthesis of a range of QS-regulated virulence factors (6,7).

The present study aimed to investigate the effects that sub-MICs of cephalosporins may have on the biofilm production of *E. coli* isolates. To investigate these effects, the biofilm production of 52 *E. coli* reference strains and clinical isolates following treatment with 1/4 MICs of third-generation cephalosporins were observed. In one *E. coli* clinical isolate, CFP and CAZ exerted opposite effects on biofilm formation. The mechanisms of these effects were then examined in that isolate.

## Materials and methods

### Bacterial strains and growth conditions

In order to investigate the effects of sub-MICs of third-generation cephalosporins on the biofilm formation of *E. coli*, at the beginning of the study each of the three *E. coli* reference strains (ATCC700926, ATCC35218 and DH5α) and 49 clinical isolates were treated separately with four third-generation cephalosporins [Ceftazidime (CAZ; Sigma-Aldrich, Shanghai, China), ceftriaxone, cefotaxime or cefoperazone (CFP) (all from National Institutes for Food and Drug Control, Beijing, China)] at 1/4 MICs. The study was approved by the Ethics Committee of Southwest Hospital, Third Military Medical University, (Chonqing, China).

The ATCC700926, ATCC35218, DH5α and BAA1117 (*Vibrio harveyi* BB170) strains were purchased from the American Type Culture Collection (Manassas, VA, USA). A total of 49 *E. coli* isolates collected from Southwest Hospital (Chongqing, China) between January 2009 and February 2009 were used in this study. Written informed consent from the patient/patient’s family was obtained prior to the study.

Among the *E. coli* examined, E42, isolated from the pus of a surgical patient who had undergone a curative resection of a colorectal carcinoma, was noteworthy as formation of its biofilm was suppressed by 1/4 MIC CAZ, while it was enhanced by 1/4 MIC CFP. To examine the underlying mechanisms controlling these opposite effects, E42 was selected for further investigation in the subsequent experiments.

*E. coli* isolates were grown at 37°C in Luria-Bertani (LB) broth, and BAA1117 was grown at 30°C in marine broth (BD, 2216). In addition, the bacterial growth was determined using LB broth containing 1/4 MIC of CAZ or CFP with rapid shaking at 37°C. For the growth curve experiments, 50 μl of the culture sample was collected every 4 h for 24 h to measure the optical density at 600 nm with a Thermo Multiskan Spectrum (Thermo Fisher Scientific, Inc., Waltham, MA, USA) (8).

### Determination of MICs for E. coli strains

The MICs of the four cephalosporins against the *E. coli* strains were determined in accordance with the Clinical and Laboratory Standards Institute guidelines (9). Cultures were adjusted to a turbidity equivalent to 0.5 MacFarlane standard suspension prior to being inoculated on Mueller Hinton agar (Oxoid, Basingstoke, UK) in the presence of CAZ, ceftriaxone, cefotaxime or CFP at concentrations ranging from 256 to 0.0625 μg/ml (12 doubling-dilution drug concentrations). Cultures were incubated for 20 h at 37°C under aerobic conditions. The lowest drug concentration that could prevent growth was recorded as the MIC.

### Biofilm formation assay

Biofilm formation was assayed by crystal violet staining of adherent cells as described previously (10), with a few modifications. The bacterial cultures that were adjusted to 1×10^7^ cfu/l were inoculated in LB broth on 96-well polystyrene plates in the presence of CAZ or CFP at sub-MICs. Following incubation at 37°C for 6–24 h, the plates were rinsed twice with phosphate-buffered saline and dried in an inverted position. The adherent cells were stained with 1% crystal violet (Sigma-Aldrich) for 10 min, and the wells were rinsed three times with sterile water. The dye was dissolved in 30% acetic acid, and the absorbance of the solubilized dye at 590 nm was then determined using the Thermo Multiskan Spectrum. Each treatment was assayed in 5-wells per plate and the experiments were repeated three times.

### Measurement of mRNA changes of the genes encoding biofilm-modulating proteins

Reverse transcription-polymerase chain reaction (RT-PCR) and quantitative (q)PCR were used to investigate the levels of the mRNA products of the biofilm-modulating genes of this isolate. The cultures were inoculated in 20 ml LB broth containing 1/4 MIC of CAZ or CFP and then cultivated at 37°C with shaking in an environmental chamber (Yuejin, Shanghai, China) for 0.5–10 h. RNA isolation was performed in accordance with the manufacturer’s instructions provided for the TRIzol reagent (Invitrogen, Carlsbad, CA, USA). RT using a Reverse Transcription system (Promega Corporation, Madison, WI, USA) was conducted as described previously (11). The primers are listed in Table I. RT-PCR and qPCR amplifications were performed. The latter were performed in quadruplet using MyiQ Color Fluorescence Real-Time Quantitative PCR apparatus (Bio-Rad Laboratories, Hercules, CA, USA). The reaction conditions are listed in Table II. For qPCR, 40 cycles were completed and the annealing temperature was 60°C. The fold changes of each transcript were calculated using the 2^−ΔΔCT^ method (12) and were expressed as values relative to the control group.

### Autoinducer-2 (AI-2) bioassay

The AI-2 bioassay was performed to determine whether AI-2 production in the E42 isolate was affected by CAZ or CFP and to verify whether the gene expression had been inhibited. The bioassays were performed in accordance with the method previously described (13,14). The strains treated with a 1/4 MIC of CAZ or CFP were pelleted by centrifugation at 12,000 × g for 10 min, and then the supernatant was filtered through a 0.22-μm filter (Millipore, Bedford, MA, USA). *V. harveyi* BB170 was diluted 1:5,000 in fresh Difco Marine Broth 2216 (BD Biosciences, Franklin Lakes, NJ, USA) and then 180 μl of culture was mixed with 20 μl of the supernatant sample. The luminescence values of the mixtures were measured with a luminometer (Turner Biosystems, Sunnyvale, CA, USA) after incubating at 30°C for 3 h. A positive control was obtained from the overnight cultures of BB170 and sterile medium served as a negative control. AI-2 activity was expressed as the difference in relative light units compared with the level of luminescence induced by the negative control. For each experiment, the bioassay was performed in triplicate for each sample.

### Transduction of antisense oligonucleotides (AS-ODNs)

AS-ODNs targeting S-ribosylhomocysteine lyase (*luxS*) of *E. coli* were designed and used to inhibit gene expression (15,16). Firstly, four AS-ODNs were designed (5′-GGTAATGGTGTAGAGATTATCGATA-3′, 5′-CAATGGAAGACGTGCTGAAAGTGCA-3′, 5′-GAACGTCTACCAGTGTGGCACTTAC-3′ and 5′-CGAAAGAGAAGTTGCAGGAACTGCA-3′; available at https://rnaidesigner.invitrogen.com/rnaiexpress/) and the one with the highest blocking efficiency was selected for the subsequent experiments. Briefly, 4 μl (20 μg) of the transfection reagent Sofast (5 mg/ml; Sunmabio, Xiamen, China) was diluted in 20 μl LB broth. Following incubation at room temperature for 20 min, 20 μl of AS-ODN was added to the diluted transfection reagent. A total of 40 μl of the mixture was added to 160 μl of the competent bacterial suspension with a final concentration of 10 μM AS-ODN. Following culturing at 37°C for 1 h, the transfected bacteria were adjusted to a turbidity equivalent of a 0.5 MacFarlane standard suspension and cultivated at 37°C with shaking in an environmental chamber for 4 h.

### Statistical analysis

The significance of the intergroup differences in terms of biofilm formation, growth or AI-2 production was analyzed using an independent samples t-test. The significance of differences in RT-PCR results was determined using a paired samples t-test. P<0.05 was considered to indicate a statistically significant difference.

## Results

### Biofilm formation by *E. coli* clinical isolate E42 is inhibited by CAZ but induced by CFP

Firstly, the effects of four third-generation cephalosporins (CAZ, ceftriaxone, cefotaxime and CFP) at 1/4 MICs on the biofilm formation of 52 *E. coli* isolates were measured. It was identified that CAZ inhibited biofilm formation in seven strains, while CFP enhanced biofilm formation in 18 strains. The results from the E42 isolate were noteworthy as biofilm formation was suppressed by CAZ at 1/4 MIC, whereas it was enhanced by CFP at 1/4 MIC. Therefore, E42 was selected for investigation in subsequent experiments.

**Figure 1:**
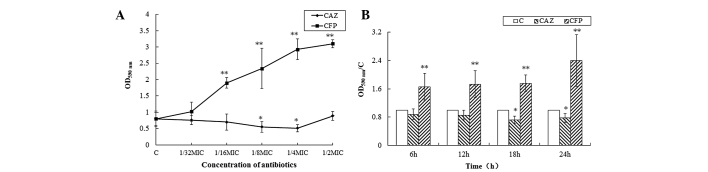
Biofilm formation of E42 in the presence of CAZ or CFP at sub-MICs. (A) Effects of 1/32 to 1/2 MICs of CAZ and CFP on biofilm formation by E42 determined by 1% crystal violet staining. (B) Effects of 1/4 MICs of CAZ and CFP on biofilm formation by E42 incubated for 6–24 h. The values are expressed as the mean ± standard deviation. All values demonstrated are from one representative experiment (n=5) of three experiments. ^**^P<0.01 and ^*^P<0.05, compared with the C, using the independent samples t-test. CAZ, ceftazidime; CFP, cefoperazone; MICs, minimum inhibitory concentrations; C, control.

CAZ at sub-MICs suppressed biofilm formation by E42 in a dose-dependent manner, and CFP enhanced its biofilm formation in a dose-dependent manner (Fig. 1A). In addition, 1/4 MIC CAZ exerted maximal effects on E42 biofilm formation at 18 h and the effect of CFP at 1/4 MIC reaches a maximum at 24 h (Fig. 1B).

### Effects of CAZ and CFP at 1/4 MICs on the mRNA expression of biofilm regulator genes

**Figure 2:**
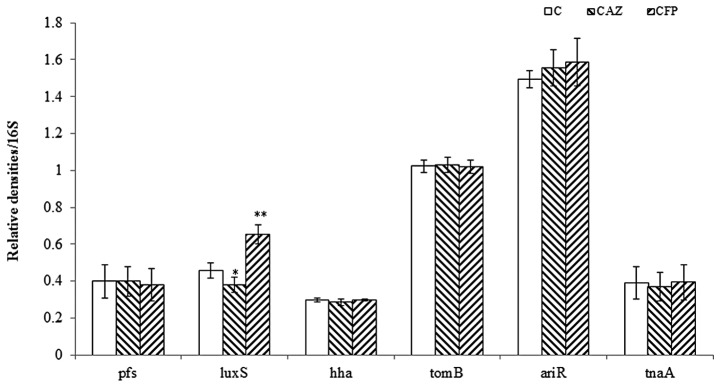
Expression of *pfs, luxS, hha, tomB, ariR* and *tnaA* in the cultures grown for 3 h in the presence of 1/4 MIC of CAZ or CFP. A 16S rRNA was used as an internal standard. For all experiments, n=3. ^*^P<0.05, compared with C. CAZ, ceftazidime; CFP, cefoperazone; MICs, minimum inhibitory concentrations; C, control; *luxS*, S-ribosylhomocysteine lyase.

To determine which regulator gene is involved in the effects of CAZ and CFP on E42 biofilm formation, the changes in the mRNA levels of important regulator genes (*pfs*, *luxS*, *ariR*, *tnaA*, *hha* and *tomb*) in the bacterial cultures 3 h following inoculation were examined. Among these six genes, the levels of *luxS* mRNA were associated with CAZ or CFP treatment, whereas mRNA levels of the five other genes were not changed (Fig. 2). The present data suggested that *luxS*/AI-2 QS may be involved in the inhibitory effects of CAZ and the inductive functions of CFP on *E. coli* biofilm formation. Therefore, the subsequent experiments focused on *luxS*/AI-2 QS.

### CAZ exerts effects opposite to that of CFP on the mRNA levels of luxS and AI-2 production in E. Coli isolates

**Figure 3:**
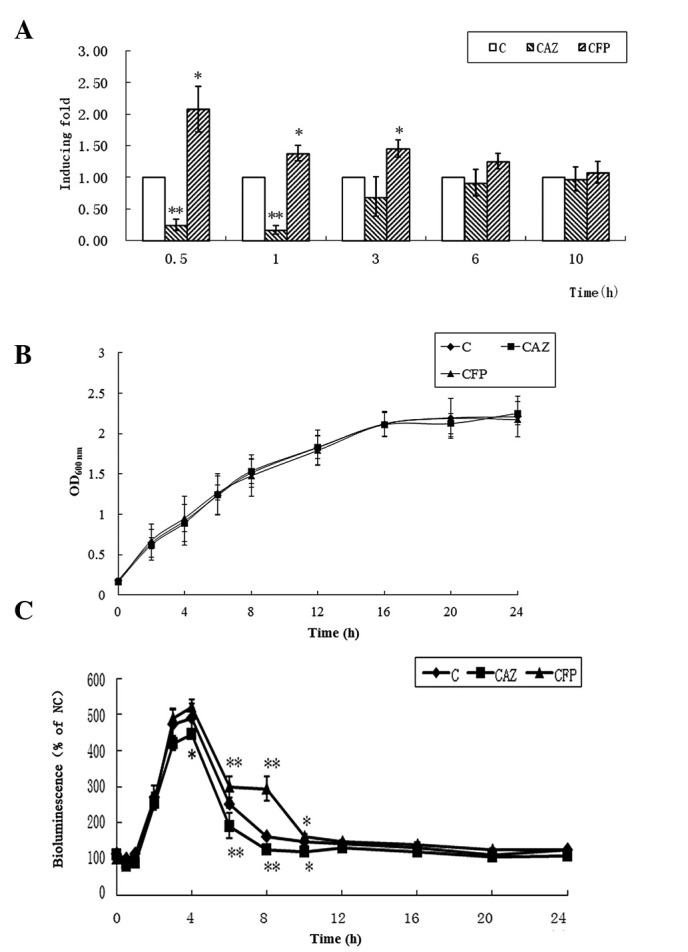
Effects of CAZ or CFP at 1/4 MIC on luxS/AI-2 QS. (A) mRNA levels of luxS determined by qPCR, (B) production of AI-2 and (C) growth of E42 in the presence of 1/4 MIC of CAZ or CFP. The values are expressed as the mean ± standard deviation. For the qPCR experiments, n=4; for the AI-2 bioassays, n=3. ^**^P<0.01 and ^*^P<0.05, compared with the C. CAZ, ceftazidime; CFP, cefoperazone; MICs, minimum inhibitory concentrations; C, control; qPCR, quantitative polymerase chain reaction; *luxS*, S-ribosylhomocysteine lyase..

QS with *luxS*/AI-2 is known to be important for *E. coli* biofilm formation (17). Therefore, the mRNA levels of *luxS* and AI-2 production in the clinical isolate E42 in the presence of 1/4 MICs of CAZ or CFP were further quantified. Considering that AI-2 production is primarily determined by the density of bacterial inoculates, the growth of bacterial cells was examined simultaneously. CAZ and CFP at 1/4 MICs exerted effects on the mRNA levels of *luxS* and AI-2 production, without any effect on the growth of the isolate (Fig. 3). In E42, the mRNA levels of *luxS* was significantly reduced following growth in the LB medium with 1/4 MIC CAZ incubated for 30 min or 1 h, compared with the controls in which CAZ was absent. However, these levels were higher following exposure to 1/4 MIC CFP for 0.5–6 h, compared with the controls without CFP (Fig. 3A). Based on the AI-2 bioassay, changes in bioluminescence occurred following treatment for 4 h (Fig. 3B). These results were concurrent with those of the mRNA levels of *luxS*.

### Effects of CAZ and CFP at 1/4 MICs on the mRNA levels of biofilm-modulating factors that are regulated by AI-2

**Figure 4:**
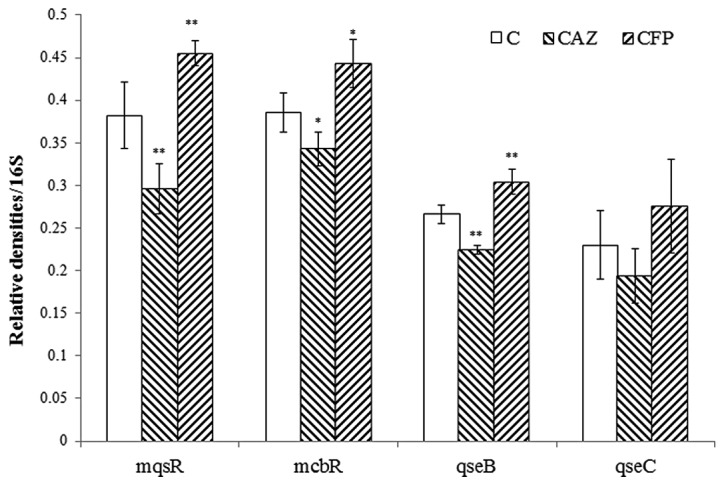
Levels of mRNA of biofilm-modulating proteins regulated by AI-2 in cultures grown for 12 h in the presence of 1/4 MIC of CAZ or CFP. 16S rRNA was used as an internal standard to allow semi-quantitative comparisons between samples. For all experiments, n=3. ^*^P<0.05, compared with C. CAZ, ceftazidime; CFP, cefoperazone; MICs, minimum inhibitory concentrations; C, control.

During biofilm formation, AI-2 stimulates biofilm formation and changes its architecture by stimulating flagella motility via MqsR, QseBC and McbR (18). The mRNA levels of key downstream genes that are regulated by AI-2 in the cultures incubated with CAZ or CFP at 1/4 MICs for 12 h. The expression levels of *mqsR*, *qseB*, *qseC* and *mcbR* in E42 were significantly decreased in the presence of 1/4 MIC of CAZ, whereas CFP exerted the opposite effect on these genes (Fig. 4).

### AS-ODNs targeting luxS modulate the effects of CAZ and CFP on E. coli biofilm formation

**Figure 5:**
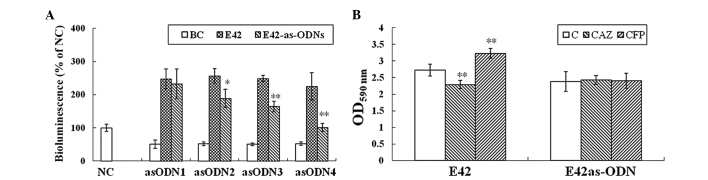
(A) AS-ODNs targeting *luxS* decrease AI-2 production and (B) the effects of CAZ and CFP on biofilm formation by *Escherichia coli* isolates. For the AI-2 bioassay experiments, n=3; ^**^P<0.01 and ^*^P<0.05, compared with E42. For the biofilm formation experiments, n=5; ^**^P<0.01 and ^*^P<0.05, compared with C. AS-ODNs, antisense oligonucleotides; CAZ, ceftazidime; CFP, cefoperazone; MICs, minimum inhibitory concentrations; AI-2, autoinducer-2; C, control.

To confirm the role of *luxS* in the response to CAZ and CFP, the AS-ODNs targeting *luxS* were employed. AI-2 production was measured to verify whether *luxS* expression was blocked. As AS-ODNs do not pass freely through cell membranes due to their negative charge, the cationic polymer reagent Sofast was used to transduce the AS-ODNs, as previously described (15,16). The results demonstrated that the transduction of AS-ODNs targeting *luxS* decreased AI-2 production significantly, indicating that *luxS* expression was successfully blocked (Fig. 5A). As AS-ODN-4 (5′-CGAAAGAGAAGTTGCAGGAACTGCA-3′) was the most efficient at blocking *luxS* expression, it was selected for the subsequent experiments. The *E. coli* biofilm formation in the presence of 1/4 MIC of CAZ or CFP was then quantified. Following AS-ODN transduction, the effects of CAZ and CFP on *E. coli* biofilm formation were diminished (Fig. 5B), suggesting that CAZ and CFP were unable to affect biofilm formation once the *luxS* gene was blocked.

## Discussion

To the best of our knowledge, this is the first study to demonstrate that two antibiotics, CAZ and CFP, sharing the same antimicrobial mechanism have opposite effects on biofilm formation of an *E. coli* clinical isolate at their respective sub-MICs.

In previous studies, drugs with similar chemical structures and antimicrobial activities have been demonstrated to have varied effects on the biofilm formation of different species of bacteria. For example, azithromycin was demonstrated to inhibit biofilm formation by *Pseudomonas aeruginosa* (19) and *Porphyromonas gingivalis* (20), but enhanced biofilm formation by *Staphylococcus epidermidis* (21). However, for a given bacterial species, it is rarely reported that drugs with similar antimicrobial activities may cause opposite changes in biofilm formation. Only one study demonstrated a similar effect, showing that clarithromycin induced biofilm formation in isolates of *Pseudomonas aeruginosa* (22), while azithromycin did the opposite (19). However, these effects took place with different strains. In the present study, it was reported that CAZ and CFP had opposite effects on biofilm formation in the same isolate of *E. coli.*

CAZ and CFP are third-generation cephalosporins. They have similar antimicrobial activities (23), based on their identical β-lactam structure. However, in the present study they induced completely opposite effects on biofilm formation in a clinical isolate of *E. coli*, indicating that these activities are likely independent of their antimicrobial roles. Furthermore, at concentrations that influenced biofilm production, these antibiotics were unable to change bacterial growth, which also suggests the independent nature of these two activities.

To examine which regulator gene is involved in the effects of CAZ and CFP on *E. coli* biofilm formation, the changes in the mRNA levels of the important regulator genes were examined, including *pfs* (encoding the 5′-methylthioadenosine/S-adenosylhomocysteine nucleosidase enzyme), *luxS* (encoding synthetases of AI-2), *hha*/*tomb* (a toxin-antitoxin pair), *tnaA* (encoding tryptophanase to synthesize indole) and *ariR* (a regulator of acid resistance influenced by indole) in 3-h bacterial cultures (18). The present results indicated that *luxS*/AI-2 QS may be associated with the effects of CAZ and CFP on *E. coli* biofilm formation. QS has been implicated in the control of a number of bacterial functions, including the secretion of virulence factors, biofilm formation, bioluminescence production, conjugation and swarming motility (2). *luxS*/AI-2 QS has been demonstrated to regulate *E. coli* biofilm formation (24). In the present study, CAZ at 1/4 MIC reduced *luxS* mRNA levels and AI-2 production in *E. coli* isolates, whereas CFP at 1/4 MIC had the opposite effect.

The designed AS-ODNs were effective at downregulating the expression of the targeted gene; AS-ODNs targeting *luxS* decreased the inhibitory effects of CAZ and the enhancer role of CFP in biofilm formation by an *E. coli* isolate. These results provide more evidence that the roles of these two compounds on biofilm formation depend on changes in QS activity.

The similar antimicrobial activities of CAZ and CFP are due to the similarity of their basic β-lactam structure (25). It was speculated that their opposite effects on biofilm formation were due to different side chains (26). To determine whether this hypothesis is reasonable, the studies published in the past three years regarding the effects of antibiotics on the biofilm formation of various bacteria were reviewed. However, there was little evidence indicating an association between biofilm-influencing activity and the structures of the antibiotics. Therefore, this hypothesis requires further investigation.

In the present study, the concentrations tested were carefully selected so that the antibiotics did not effect the bacterial growth. Nevertheless, the biofilm formation of the *E. coli* isolate E42 was affected by these low CAZ and CFP doses. Previous studies have demonstrated that the effects of certain antibiotics on the growth and virulence-gene expression of bacteria at higher concentrations than MICs may act via QS (6,17). The results indicate that the response of this isolate to CAZ and CFP at 1/4 MIC may also be due to QS.

In the present study, the biofilm formation of 52 *E. coli* isolates was measured, but only the production of biofilm by E42 responded to CAZ in an opposite manner to that of CFP. This result appears to indicate an individual case. Differences in the reactions among *E. coli* isolates appear to be due to individual differences in isolates. The QS of *luxS*/AI-2, as an important intermediate, may be involved in the responses to CAZ and CFP, while the underlying mechanisms require further study.

In conclusion, the present study indicates that the effects of antibiotics at sub-MICs may differ from their effects at high doses. The present study does not fully explain the potential effects of these drugs, and therefore extensive and careful investigation is required to determine the clinical application of this data.

## Figures and Tables

**Table I:** Oligonucleotides used in reverse transcription-polymerase chain reaction.

Gene	Accession no.	Primer	Sequence (5′-3′)	Product size (bp)
*16s-rRNA*	NC_000913	16s-rRNA-F	ggaggaaggtggggatgacg	638
		16s-rRNA-R	atggtgtgacgggcggtgtg	
*pfs*	NC_000913	pfs-F	cctggcaccaacgttgaaag	347
		pfs-R	tggcgcgtacgacaacaaac	
*luxS*	NC_000913	luxS-F	tgccgaacaaagaagtgatgc	348
		luxS-R	cttcgttgctgttgatgcgtac	
*ariR*	NC_000913	ariR-F	tcagcagtgttagggcaggc	156
		ariR-R	tcgcaacacgatttccagtg	
*hha*	NC_000913	hha-F	aatgcgtttacgtcgttgcc	135
		hha-R	ttcatggtcaattcggcgag	
*tnaA*	NC_000913	tnaA-F	aactgttgccgcatatcccg	248
		tnaA-R	attcgccgcgttctctttca	
*tomB*	NC_000913	tomB-F	caatcatggctgggtaaacga	265
		tomB-R	cgcaggattctctttcgtcg	
*mcbR*	NC_000913	mcbR-F	cgctttctgtcgcacctgca	191
		mcbR-R	gcccttttcttgcgcctgct	
*mqsR*	NC_000913	mqsR-F	caatgccgggcaagttcgta	186
		mqsR-R	tggcctgtaacaagcctggg	
*QseB*	NC_000913	qseB-F	cgtcagggaaaagaggcgct	258
		qseB-R	ggttcggcgcatcagagctt	
*QseC*	NC_000913	qseC-F	actcgcgccgctgaacaaac	273
		qseC-R	agtgcttttttccgcgcctg	

F, forward; R, reverse; *luxS*, S-ribosylhomocysteine lyase.

**Table II:** Polymerase chain reaction protocols.

	Reaction conditions				
	
Gene	Denaturation	Annealing	Extension	Final extension	No. of cycles
*16s-rRNA*	94°C for 30 sec		72°C for 30 sec	72°C for 10 min	30
*pfs*	94°C for 30 sec	53°C for 30 sec	72°C for 30 sec	72°C for 10 min	30
*luxS*	94°C for 30 sec	57°C for 30 sec	72°C for 30 sec	72°C for 10 min	30
*airR*	94°C for 30 sec	52°C for 30 sec	72°C for 30 sec	72°C for 10 min	30
*hha*	94°C for 30 sec	52°C for 30 sec	72°C for 30 sec	72°C for 10 min	30
*tnaA*	94°C for 30 sec	54°C for 30 sec	72°C for 30 sec	72°C for 10 min	30
*tomB*	94°C for 30 sec	51°C for 30 sec	72°C for 30 sec	72°C for 10 min	30
*mcbR*	94°C for 30 sec	57°C for 30 sec	72°C for 30 sec	72°C for 10 min	30
*mqsR*	94°C for 30 sec	55°C for 30 sec	72°C for 30 sec	72°C for 10 min	30
*qseB*	94°C for 30 sec	55°C for 30 sec	72°C for 30 sec	72°C for 10 min	30
*qseC*	94°C for 30 sec	56°C for 30 sec	72°C for 30 sec	72°C for 10 min	30
